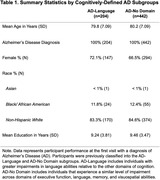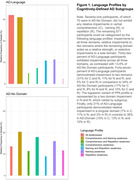# Characterization of Language Profiles in Cognitively‐Defined Subgroups of Alzheimer’s Disease

**DOI:** 10.1002/alz.084033

**Published:** 2025-01-03

**Authors:** Jeanne Gallée, Laura E. Gibbons, Shubhabrata Mukherjee, Phoebe Scollard, Seo‐Eun Choi, Bryan D James, Brandon S Klinedinst, Michael L. Lee, Jesse Mez, Emily H. Trittschuh, Andrew J. Saykin, Paul K. Crane

**Affiliations:** ^1^ University of Washington School of Medicine, Seattle, WA USA; ^2^ University of Washington Alzheimer’s Disease Research Center, University of Washington School of Medicine, Seattle, WA USA; ^3^ University of Washington, School of Medicine, Seattle, WA USA; ^4^ University of Washington, Seattle, WA USA; ^5^ Department of Internal Medicine Rush University Medical Center, Chicago, IL USA; ^6^ Rush Alzheimer’s Disease Center, Chicago, IL USA; ^7^ Boston University Chobanian & Avedisian School of Medicine, Boston, MA USA; ^8^ VA Puget Sound Health Care System, Seattle Division, Seattle, WA USA; ^9^ UW School of Medicine, Seattle, WA USA; ^10^ Indiana Alzheimer’s Disease Research Center, Indianapolis, IN USA; ^11^ Department of Medical and Molecular Genetics, Indiana University School of Medicine, Indianapolis, IN USA; ^12^ Indiana Alzheimer’s Disease Research Center, Indiana University School of Medicine, Indianapolis, IN USA

## Abstract

**Background:**

The relationship between Alzheimer’s disease (AD) pathology and the associated clinical syndrome a patient presents with remains indeterminate. Cognitively‐defined subgroups of AD have revealed distinctions based on relative cognitive impairments, including AD‐Language, where challenges in language are substantial, and AD‐No Domain, where no relative asymmetries across cognitive domains occur. Pathological features of AD have been associated as the primary neuropathology of the logopenic variant of primary progressive aphasia (lvPPA). Hallmark clinical features of lvPPA include relatively spared comprehension in the face of decline in naming and repetition abilities. This work aimed to test the hypothesis that the lvPPA language profile was overrepresented in AD‐Language when compared to AD‐No Domain.

**Method:**

Measures of verbal comprehension, confrontation naming, and phrase‐level repetition were obtained from all participants from the Religious Orders Study (ROS), the RUSH Memory and Aging Project (MAP) and the Minority Aging Research Study (MARS) using confirmatory factor analyses. We subsetted the data to include participants belonging to the AD‐Language and AD‐No Domain groups at their initial AD diagnosis visit. We compared patterns of language profiles based on strengths and weaknesses in comprehension, naming, and repetition. Pearson’s Chi‐squared tests with Yates continuity correction was used to test if the language patterns were statistically different between the two AD subgroups.

**Results:**

We analyzed language performance in 642 participants across AD‐Language (31.8%) and AD‐No Domain (68.2%) groups (Table 1). Thresholds were based on AD‐No Domain and set as the median for each subdomain (comprehension = ‐.101, naming = ‐.957, repetition = .233) to establish whether a score represented a relative strength or weakness in the language profile. Eight patterns of language profiles based on strengths and weaknesses in comprehension, naming, and repetition were formed (Figure 1). The distribution of language patterns differed significantly between AD‐Language and AD‐No Domain (χ2 = 97.6, p <.001). Furthermore, the lvPPA pattern was found more frequently in AD‐Language (χ2 = 28.1, p <.001).

**Conclusion:**

Heterogeneity within the AD‐Language spectrum includes a significant proportion that is consistent with the language profile of lvPPA. Relative performance in domains of verbal comprehension, confrontation naming, and phrase‐level repetition varied by AD subgroup.